# The Relationship between Nurses’ Training and Perceptions of Electronic Documentation Systems

**DOI:** 10.3390/nursrep11010002

**Published:** 2021-01-01

**Authors:** Nohel Zaman, David M. Goldberg, Stephanie Kelly, Roberta S. Russell, Sherrie L. Drye

**Affiliations:** 1 Department of Information Systems and Business Analytics, Loyola Marymount University, Los Angeles, CA 90045, USA; nohel.zaman@lmu.edu; 2 Department of Management Information Systems, San Diego State University, San Diego, CA 92182, USA; 3 Department of Business Education, North Carolina A&T State University, Greensboro, NC 27411, USA; sekelly@ncat.edu (S.K.); sldrye@ncat.edu (S.L.D.); 4 Department of Business Information Technology, Virginia Tech, Blacksburg, VA 24060, USA; rrussell@vt.edu

**Keywords:** electronic documentation, electronic medical records, technology acceptance model, nurse training, general computer skills, self-efficacy

## Abstract

Electronic documentation systems have been widely implemented in the healthcare field. These systems have become a critical part of the nursing profession. This research examines how nurses’ general computer skills, training, and self-efficacy affect their perceptions of using these systems. A sample of 248 nurses was surveyed to examine their general computer skills, self-efficacy, and training in electronic documentation systems in nursing programs. We propose a model to investigate the extent to which nurses’ computer skills, self-efficacy, and training in electronic documentation influence perceptions of using electronic documentation systems in hospitals. The data supports a mediated model in which general computer skills, self-efficacy, and training influence perceived usefulness through perceived ease of use. The significance of these findings was confirmed through structural equation modeling. As the electronic documentation systems are customized for every organization, our findings suggest value in nurses receiving training to learn these specific systems in the workplace or during their internships. Doing so may improve patient outcomes by ensuring that nurses use the systems consistently and effectively.

## 1. Introduction

Using electronic documentation systems such as electronic health records (EHRs) and electronic medical records (EMRs) reduces medical errors that promote quality, efficiency, and safety of medical care by providing the correct information to treat patients in a timely manner [1,2,3,4]. EMRs provide a digital version of a patient’s medical chart, while EHRs extend beyond this to also include records from additional doctors, providing a more long-term and holistic picture of patient health. Past research shows that using electronic health information technologies leads to reductions in errors [5]. The use of electronic documentation systems can improve overall clinical documentation completeness, legibility, and understandability when compared to traditional paper-based medical records [6]. As hospitals increasingly seek to make data-driven decisions [7], the use of these systems has become paramount.

In the health care profession, nurses obtain and retrieve most of the information in a patient’s EHR [1]. Kim et al. [8] found that nurses spend up to 25% of their time on documentation. Nursing documentation serves various purposes, such as collecting data on a patient’s health condition or illness for planning and evaluating care, communication among health care professionals, research, public health management, norms and standards development, and providing evidence for legal issues [8]. The quality of the documentation is often considered to be a reflection of the quality of the care provided [8]. Thus, nurses’ proficiency with electronic documentation systems is a critical component of effective patient care.

Recent research suggests value in developing nursing education for building technology skills of nursing students [9,10]. However, there is a shortage of research that examines nurses’ technology training, whether for general IT skills (word processing, email, database searches, etc.) or for other skills related to working with electronic documentation systems. The primary focus of this study is to examine how general computer skills and electronic documentation system training affect nurses’ perceptions of using electronic documentation systems. If it is found that nurses perceive that they need more general computer skills, information systems professionals are in a good position to offer basic computer classes that would be beneficial for nursing students. In order to investigate factors influencing nurses’ acceptance of electronic documentation systems in nursing practice, the technology acceptance model (TAM) [11] conceptual framework will be used.

The proposed model has been modified to develop TAM in the context of an electronic documentation system used by nurses in clinical practice. The modified model describes nurses’ computer skills, training and self-efficacy in electronic documentation systems that could affect their perceptions in using these systems. Gagnon et al. [12] tested computer self-efficacy influencing physicians’ intention to use EHR and found that physicians were more likely to accept the technology if they felt that it was easy to use. In our model, training to use an electronic documentation system has been added along with computer skills for testing the hypotheses that they affect perceived ease of use in EHR. Compared to models of prior studies [12,13], this model compares the predictive performance of the external factors: nurses’ computer skills, electronic documentation system training, and its self-efficacy to investigate their effects on nurses’ perceptions of using electronic documentation systems.

## 2. Conceptual Model and Research Hypotheses

**Figure 1:**
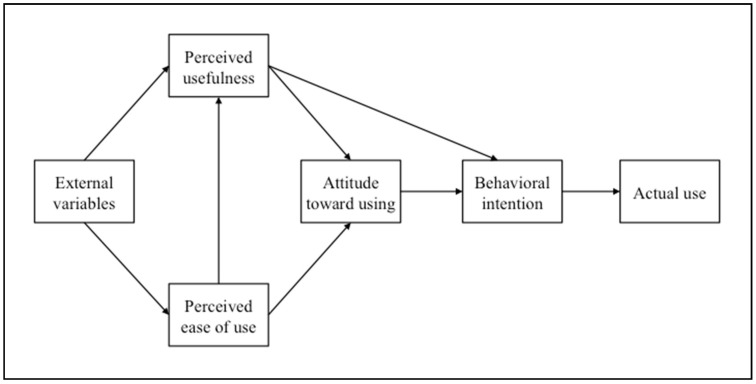
Original technology acceptance model (adapted from Davis et al. [11], p. 985).

The technology acceptance model (TAM) was first introduced in the 1980s and has widely been used by researchers to study technology acceptance [14]. TAM provides a foundation to investigate how external variables affect intention to use a particular technology. The original TAM (see Figure 1) has five key determinants: perceived ease of use (PEOU), perceived usefulness (PU), attitude toward using (ATU), behavioral intention to use (ITU), and actual use (AU) of a computer system [14,15,16]. External variables such as social factors affect individuals’ initial perceptions of new technologies and influence PU (the degree to which an individual believes the technology will improve their performance) and PEOU (the degree to which an individual believes the technology will be low-effort to use). These factors subsequently affect attitudes toward technology and ultimately predict actual use.

Researchers have utilized TAM to investigate how medical professionals and patients accept and use various kinds of technology, such as EHRs [17], software products [18], mobile information technology [19,20,21], and telemedicine technology (eICU) [16]. Additionally, previous studies have used TAM as a theoretical framework to explore the students’ acceptance of technology in educational settings, such as web-based learning, online courses, and clinical imaging portal for developing healthcare education [22,23,24]. Kowitlawakul et al. [13] found that nursing students with more positive attitudes towards EHRs in education were more likely to believe that the systems were easy to use.

### 2.1. Proposed Constructs and Definitions

For this study, TAM will be utilized with constructs of self-efficacy, general computer skills, and training on electronic documentation system to study nurses’ technology training and how it affects their intentions to use EHRs. TAM’s constructs of perceived usefulness, perceived ease of use, and behavioral intention to use will also be examined in this study with respect to EHRs. The proposed TAM constructs and definitions are detailed in Table 1.

The proposed model incorporates the external variables (i.e., self-efficacy, training, and general computer skills) and the key determinants (i.e., PEOU and PU) of the original TAM to evaluate their effects on the nurses’ intentions to use the electronic documentation systems. The external variables and the key determinants are chosen in this study because the evidence in previous studies [11,14] show a significant relationship on the behavioral intention to use the electronic documentation systems.

### 2.2. Research Hypotheses

In the following, we describe the research hypotheses pertaining to each construct in our model. We propose an adaptation of TAM to consider nurses’ acceptance of electronic documentation systems.

General computer skills have been studied in prior literature [26,27] and are included in the proposed model to investigate their coverage in the nursing curriculum and whether they extend to which they affect the perceived ease of use or usefulness of an electronic documentation system. In a study about nursing students’ attitudes, the students reacted positively to learning about technology, even though neither IT curriculum was offered nor did the students have option to explore IT application-related courses [26]. The study also found low exposure to IT education, even in graduate-level nursing schools [26]. Nurses’ computer competency (i.e., computer knowledge, computer attitudes, and computer skill) has been studied previously [29], and general computer skills in the current study relate specifically to knowledge such as word processing, spreadsheets, Internet searches, and email. However, research has yet to examine whether nurses who report having good general computer skills would perceive an electronic medical record system to be useful and easy to use, as those variables ultimately determine their behaviors. For example, would having general computer skills help a nurse to feel more confident in being able to use a system? Would general computer skills enable a nurse to better determine if a system is easier to use or to feel more confident in using it? Thus, the following hypotheses are proposed:

**Hypothesis 1a** **(H1a).** *General computer skills of nurses positively influence the perceived usefulness of electronic documentation systems*.

**Hypothesis 1b** **(H1b).** *General Computer skills of nurses positively influence the perception of electronic documentation systems’ ease of use*.

Past studies have demonstrated that self-efficacy predicts the use of new technologies [30]. Computer self-efficacy [24,25] is defined as an individual’s perception of his or her own ability to use a computer in the accomplishment of a task. Computer self-efficacy incorporates judgments about the ability to apply skills to broader tasks, such as preparing written reports or analyzing financial data [24].

Without skills, performance is not effective; without self-efficacy, performance may not be attempted [19,30]. Previous studies in the context of nursing education have shown that computer self-efficacy exhibited a strong indirect effect on behavioral intention to use technology [23,24]. Several studies have empirically shown that self-efficacy affects perceived usefulness as well as perceived ease of use [11,19,31]. The following relationships are hypothesized in the context of self-efficacy in using an electronic documentation system:

**Hypothesis 2a** **(H2a).** *Self-efficacy of nurses positively influences the perceived usefulness of electronic documentation systems*.

**Hypothesis 2b** **(H2b).** *Self-efficacy of nurses positively influences the perception of electronic documentation systems’ ease of use*.

Training is a crucial factor in new information technology acceptance because theory and evidence assert that individual perceptions in new information technology acceptance may increase over time with sufficient technical support and training programs [21]. However, Wu et al. [21] found that technical support and training have no significant effects on both perceived usefulness and perceived ease of use, and the researchers suggested that the majority of information technologies should be designed to be user friendly with an intuitive interface, which considerably improves the functionality of systems, particularly ease of ease. Additionally, Holden et al. [17] predicted that the effect of training is indirect, mediated through perceived ease of use and/or perceived usefulness. The authors mentioned that the facilitating conditions, such as training and support and demographic variables such as age and experience, may still be important predictors in recent technology acceptance studies with healthcare professionals when using healthcare information technology. Researchers have found a lack of in-service training in electronic nursing documentation in the classroom and in practice [32]. Although there is a high rate of implementation of EHRs established in healthcare organizations in the US, very little published research exists regarding the most effective way to train nurses in the use of an EHR system or nurses’ attitudes toward EHRs for providing patient care [33].

To facilitate electronic documentation systems, it is essential to have a better understanding of what nurses need to improve through training. Research shows that valuable training programs will efficiently increase individual capabilities and their perceptions of using new information technology [21]. Consequently, training has been anticipated to make nurses more comfortable with the electronic documentation context as well as increasing their confidence in handling the new healthcare information technology. Therefore, the following hypotheses are proposed:

**Hypothesis 3a** **(H3a).** *Training attained by nurses positively influences the perceived usefulness of electronic documentation systems*.

**Hypothesis 3b** **(H3b).** *Training achieved by nurses positively influences the perception of electronic documentation systems’ ease of use*.

Perceived ease of use is defined as the “degree to which a person believes that using a particular system would be free of effort” [15]. Davis [15] stated that all else being equal, a system perceived to be easier than another is more likely to be accepted by users. Previous studies found that perceived ease of use had a significant positive effect on perceived usefulness [15,22]. In the context of examining perceptions of using electronic documentation system by nurses in this study, the following hypotheses are proposed:

**Hypothesis 4a** **(H4a).** *Nurses’ perception of electronic documentation systems’ ease of use positively influences nurses’ intention to use the electronic documentation systems.*

**Hypothesis 4b** **(H4b).** *Nurses’ perception of electronic documentation systems’ ease of use positively influences nurses’ perceived usefulness of electronic documentation systems.*

Perceived usefulness is defined as “the degree to which a person believes that using a particular system would enhance his or her job performance” [15]. Davis [15] stated that a system high in perceived usefulness is one for which a user believes in the existence of a positive use-performance relationship.

Davis et al. [11] argue for a positive relationship between perceived usefulness and behavioral intention based on the idea that users develop intentions toward behaviors that they think will increase the performance of their task. Previous research exploring public health nurses’ intentions toward using web-based learning found that perceived usefulness showed a significant direct impact on behavioral intention [22]. Intentions towards behaviors of end-users are based mainly on cognitive decision rules to enhance performance [15]. In order to explore perceptions of using electronic documentation system in this study, the following hypothesis is proposed:

**Hypothesis 5** **(H5).** *Nurses’ perceived usefulness of electronic documentation systems positively influences nurses’ intention to use electronic documentation systems*.

### 2.3. Proposed Model

**Figure 2:**
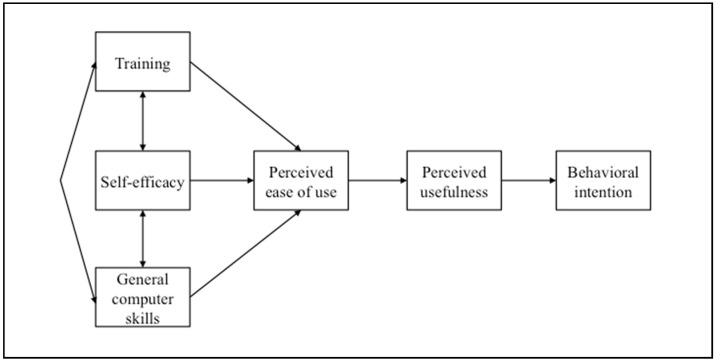
Proposed model.

As attitude has been found to only partially mediate the effects of perceived usefulness and perceived ease of use on behavioral intention [28], it is not included in our proposed model. However, it is anticipated that a mediated effect will be observed such that PEOU and then PU mediate the exogenous variables and then behavioral intention. Thus, the proposed model is displayed in Figure 2. Our model suggests that training, self-efficacy, and general computer skills are all interrelated and principally relate to an individual’s PEOU. Then, PEOU is related to PU and, finally, the individual’s behavioral intention.

## 3. Research Methodology and Descriptive Statistics

An online IRB-approved (protocol ID number: 15-0085; date of approval: 04/01/2015; IRB institution: North Carolina A&T State University) questionnaire was distributed to nurses in the hospitals within the United States to empirically assess our model. A Qualtrics agent was used to distribute this survey and obtain the responses utilized in this research. The participants were nurses currently working with electronic documentation systems. The survey consisted of 63 questions. A total of 248 nurses fully completed the online survey. We also received 30 partial responses, but we only include the 248 full responses in our ensuing analyses.

### 3.1. Participants

The majority of the nurses surveyed were females (93.5%), and most were aged between 42 and 64, consistent with national nursing demographics [34]. Table 2 summarizes the demographic characteristics of the nurses, along with their response regarding the requirement to use EHRs during their nursing practice.

### 3.2. Instrumentation

In the following, we describe the survey instruments used to assess each construct in our model. Each scale discussed is drawn from prior validated literature and was utilized as a part of our model.

#### 3.2.1. General Computer Skills

General computer skills were assessed with the measure proposed by Hobbs [29]. The measure consisted of 14 Likert items, with each response scale ranging from 1 (disagree) to 6 (agree). Moody et al. [35] reported sound to construct validity and reliability when using this measure.

#### 3.2.2. Self-Efficacy

Self-efficacy in using electronic documentation systems was measured with the assessment of Venkatesh and Davis [31], which is composed of 8 Likert items with 6-point response scales ranging from 1 (disagree) to 6 (agree). The measure is reported to have adequate reliability and construct validity [13].

#### 3.2.3. Training

Training to use electronic documentation systems was measured with the assessment of Holden, Brown [17], which is used composed of 10 Likert items with 6-point response scales ranging from 1 (disagree) to 6 (agree). Holden et al. [17] reported a high reliability score and adequate content validity.

#### 3.2.4. Perceived Ease of User

The perceived ease of using [15] electronic documentation system measure consisted of 11 Likert items, with each response scale ranging from 1 (disagree) to 6 (agree). Park and Chen [19] and Chow et al. [23] found high reliability scores. Additionally, Davis [15] reported moderate convergent, discriminant, and content validity of the perceived ease of use scales.

#### 3.2.5. Usefulness

Perceived usefulness [15] of electronic documentation system is composed of 15 Likert items with 6-point response scales ranging from 1 (disagree) to 6 (agree). Park and Chen [19], Chow et al. [23], and Wu et al. [36] reported high reliability for this measure. Further, Davis [15] reported strong convergent and discriminant validity.

#### 3.2.6. Behavioral Intention

Behavioral intention [25] to use electronic documentation system was assessed with four Likert type items, with each response scale ranging from 1 (disagree) to 6 (agree). Chow et al. [23] reported a high reliability score. Holden et al. [17] also reported the measure to have adequate convergent and discriminant validity.

### 3.3. Sample Descriptive Statistics

Table 3 below reports descriptive statistics pertaining to our sample of 248 respondents.

## 4. Results and Model Testing

Notably, only three of the nurses surveyed were not required to use EHRs (see Table 3). This skewness in the sample is most clear in the measure’s skewness of −4.68 and kurtosis of 31.17 (see Table 3). As such, ITU was excluded from the proposed model, as this requirement would likely have interfered with nurses’ autonomous intents.

Confirmatory factor analysis (CFA) was used to evaluate each unidimensional measurement model, testing for both internal consistency and parallelism using the AMOS maximum-likelihood parameter estimation algorithm. The AMOS program can provide an analysis of fit index values that can be used to examine the model fit for the data collected [12]. Testing for internal consistency allows weak or less obvious problematic items that threaten the validity of the measure to be identified and removed. Every time an item was removed from a measurement model, it was re-specified without the problematic item.

Next, CFA required a test for parallelism. This ensures that all items use assess only one construct in a set of measures. Thus, items that reflect more than one construct can be identified and removed. We dropped the items that caused a statistically significant amount of residual error until we had measurement statistics (see Table 4) of GFI greater than or equal to 0.90 and RMSEA less than or equal to 0.07.

Changes were made to the following items:

- 7 items dropped from GCS (out of 14 items);
- 2 items dropped from SE (out of 8 items);
- 6 items dropped from T (out of 10 items);
- 3 items dropped from PEOU (out of 11 items);
- 3 items dropped from PU (out of 15 items).

The items that were included under each of the constructs are given in Table 5, and the items that were excluded are shown in Appendix A.

### 4.1. Hypotheses Testing

Most of the hypotheses were strongly supported except for hypotheses H1a, H2a, and H3a. The hypotheses H4a and H5 were not tested as all the items of the measure, behavioral intention (ITU), was dropped due to sampling as discussed above. Data indicates that GCS had a direct effect on PEOU (H1(b): β = 0.33, *p* < 0.05). While SE had a significant direct impact on PEOU (H2b: β = 0.31, *p* < 0.05), it had an indirect effect on PU through the mediator of PEOU; meanwhile, PEOU had an extremely strong effect on PU (H4b: β = 0.66, *p* < 0.05). Additionally, T had a direct significant impact on PEOU (H3b: β = 0.36, *p* < 0.05). Table 6 presents correlation coefficients for the measured variables.

### 4.2. Model Testing

**Figure 3:**
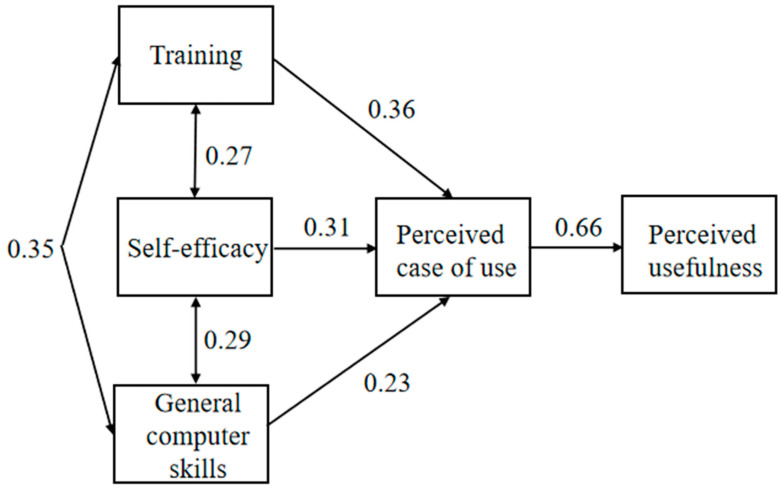
Observed model.

Figure 3 presents the standardized path coefficients that refer to the significant structural relationship among the tested variables. Because of the exclusion of ITU, the predicted model must be re-specified. The path model predicted that general computer skills, self-efficacy, and training would induce perceived ease of use, which would, in turn, induce perceived usefulness. As such, it was predicted that perceived ease of use would mediate the relationships between the proposed exogenous variables (GCS, SE, and T) and the perceived usefulness. Structural equation modeling using the AMOS maximum-likelihood parameter estimation algorithm was used to test this model. The hypotheses and path coefficients for this tested model are reported in Figure 3.

As proposed, the model fits with a chi-squared value of 3.58, the goodness of fit index (GFI) of 0.99, and root mean square error approximation (RMSEA) of 0.03. Thus, the measurement model had a good fit as the GFI was greater than 0.90, and the RMSEA is less than 0.06 [37].

### 4.3. Supplemental Analysis

We tested on differences between age groups based on the exogenous factors general computer skills (GCS), self-efficacy (SE), and training (T) by using Tukey’s HSD test. A one-way ANOVA was run for each of the exogenous variables to see if age impacted any of these traits. There is a statistically significant difference between the SE of nurses in the 58–64 group and the 18–25, and 26–33, and 34–41 groups such that the 58–64 group had lower SE. As for GCS, there is a statistically significant difference between the 50–57 and 58–64 groups versus the 18–25 and 26–33 groups such that the 50–57 and 58–64 groups had lower GCS. In addition, the over 64 group was statistically significantly different than the 26–33 group, such that the over 64 group was lower. As for Training, there was no statistically significant difference observed between age groups.

## 5. Discussion and Implications

The data were consistent with perceived ease of use mediating the relationships between proposed external variables (i.e., general computer skills, self-efficacy, and training) and perceived usefulness. Every construct in the final model was predicted to correlate positively with the others. The effects observed in the new model were consistent with expectations. Further, the effects were consistent with perceived ease of use, acting as a mediating variable between the exogenous variables and perceived usefulness.

That SE was the most influential exogenous variable has implications for nurse training. This finding suggests that the more self-confident a nurse is in using the electronic documentation system, the more useful the nurse will perceive the system at work. Furthermore, these findings were supported by nurses’ responses to open-ended questions regarding using electronic documentation systems, such as: “Yes, we had training sessions, but I learned more by actually using it in practice.” As nurses were working with the system daily, they were becoming efficient in using the system by their own ability.

### 5.1. General Computer Skills (GCS) and Perceived Usefulness (PU)

Results showed that nurses’ general computer skills were not related to the perceived usefulness of electronic documentation systems. The observed effect was inconsistent with the mediation effect. This finding resonated with the responses of nurses from the qualitative data. One of the nurses commented that nurses should know: “basic program information such as how to save and close documents. It is very interesting how little some of the new employees at my hospital know. Having to learn things like “double-click” while they’re trying to learn the electronic documentation system and hospital policies make those new employees very discouraged.”. As per this nurse’s comment, some nurses did not attain their computer skills during their nursing education, and so they had a hard time learning to use the electronic documentation system in their new jobs. However, since electronic documentation systems are mandatory for use, it may be assumed by nurses that the system must be useful.

### 5.2. General Computer Skills (GCS) and Perceived Ease of Use (PEOU)

Findings indicated that nurses’ general computer skills were related to perceived ease of using the electronic documentation system. Hobbs [29] argued that nurses who are skilled in the use of computer technology (e.g., word processing, use of tables and graphs, email) have a positive attitude in using any equipment and software that are used in their job environment. Hence, this finding coincides with the implications of previous studies as Hobbs [29] suggested that a computer competent nurse has the ability to effectively use the computer systems available and adapt his or her use to a variety of particular settings. As general computer skill weakly predicted for perceived easiness in using the electronic system, this finding also agreed with several nurses’ comments:

- “A good understanding of basic understanding programs would be helpful prior to EMR training”;
- “They need a decently strong background in computer skills. I use 6 different computer programs at work…”;
- “I did not learn my computer skills from nursing degree programs. I learned my computer skills through schooling that I took after my nursing degree. I believe it should be taught during nursing degree programs because it is very useful ay work. We always use computer skills every day. Some nurses have a hard time doing it, especially the older nurses who did not have the proper training.”

### 5.3. Self-Efficacy (SE) and Perceived Usefulness (PU)

Results indicated that self-efficacy in electronic documentation systems did not relate to perceived usefulness. Abdullah and Ward [38] found that there is a lack of association between self-efficacy and perceived usefulness. However, Kowitlawakul et al. [13] found that self-efficacy explained 29 percent of the variance in perceived usefulness of electronic health records for nursing education. Yet, in our study, 99 percent of the nurses that were surveyed are required to use the electronic documentation system in their job.

### 5.4. Self-Efficacy (SE) and Perceived Ease of Use (PEOU)

Among the most crucial among the findings was that self-efficacy was a strong predictor for perceived ease of use. The results indicated that nurses who had higher self-efficacy in using electronic documentation systems were more likely to believe that the system is easy to use. In a broader context, self-efficacy with computers in general also seemed to be important. Nurses noted in the open-ended questions that, “Being confident in the use of computers will help,” and “I think teaching people not to be afraid of computer use is important.” One nurse noted that self-efficacy to work in an evolving environment is also important: “This is an area that is still in the making. Just teach nurses to be prepared for change as well as not to rely on the computer for hands-on knowledge, so when the system goes down, which happens, nurses do not feel like they cannot do their jobs.”

In the open-ended responses, nurses also stated that there were too many types of current electronic documentation systems to be able to learn them all. In addition, many were customized for each practice and even for each specialty area. A number of nurses stated that when they received formal training in using the electronic system, they continued to learn more on the job that allowed them to become proficient. This suggested that a mixture of training and everyday usage of the system built a nurse’s self-efficacy in using the electronic documentation system because the systems can be very complicated.

Nurses’ self-efficacy is increased when learning to use the system every day in their jobs. This suggests that the application of the systems should be designed in a simple, consistent way so that the nurses find it very easy to navigate as well as document patients’ records electronically when they get to use the system every day.

### 5.5. Training (T) and Perceived Usefulness (PU)

The current results verified Wu et al. [21]’s predictions related to MHS training that training in electronic documentation systems did not relate to the perceived usefulness of the system. In the open-ended questions, nurses commented: “Since there are several main systems, it would be difficult to train most students on the specifics of one or two programs” and “There are so many different systems that it could be hard to train.”. The comments suggested that if there was training offered to nurses for learning about a particular system during their education, they would not still experience its usefulness as they may need to use a different system in their job. Since the survey data (Supplemental Materials) showed that 99 percent of nurses mandatorily used the system every day, training was not necessary for them to promote the usefulness of the system.

### 5.6. Training (T) and Perceived Ease of Use (PEOU)

Results showed that training was related to perceived ease of use in electronic documentation systems, whereas Wu et al. [21] found that training had no impact on perceived ease of use in the context of mobile computing. Our finding suggests that nurses’ training to use an electronic documentation system was not the major factor for the nurses to easily use the system in their job environment. Additionally, our results regarding training to use the system imply that nurses’ training to use the electronic documentation system moderately influences nurses’ perceived ease of use of the system. There was an insufficient level of training being offered to nurses according to the open-ended responses:

- “Should be taught the data elements that need to be captured. Since software varies from facility to facility, in-house training should cover where the documentation elements are captured and explain that free texting usually isn’t searchable.”
- “All nurses should be familiar with e-charting. It is definitely the present and the future in nursing documentation. If a hospital is still using paper charting, they are an exception to the rule. It’s imperative that nurses are able to chart electronically in all aspects of nursing.”
- “All nursing students should receive EMR training that precisely mirrors the training their nursing preceptors received on their clinical sites. If a student cannot effectively document or find information on their patients in the EMR, they may jeopardize the care of their patients. There are too many EMRs to be able to educate all nursing students on each one and have them be proficient.”

### 5.7. Perceived Ease of Use (PEOU) and Perceived Usefulness (PU)

The results indicate that perceived ease of use largely influences electronic documentation systems’ perceived usefulness because approximately 66 percent of variance was explained from perceived ease of use for perceived usefulness. This may imply that nurses’ feelings about the electronic documentation systems’ ease of use are the most influential factor on its perceived usefulness. One of the responses to an open-ended question was, “All students should be familiar with e-charting. It is definitely the present and the future in nursing documentation. If a hospital is still using paper charting, they are an exception to the rule. It is imperative that student nurses are able to chart electronically in all aspects of nursing.”

However, in a previous study, physician’s feelings about smartphone usefulness played a more influential factor than physician’s perceptions of easiness in determining physicians’ attitude toward using it [19]. Wu et al. [36] mentioned that healthcare professionals’ perceived ease of use had a very weak impact on perceived usefulness. Nurses’ perceived ease in using mobile information technology also had a moderate impact on the technology’s perceived usefulness [20]. Nursing students’ perceived ease of using electronic health records moderately influences its perceived usefulness, according to Kowitlawakul et al. [13]. Hence, the current findings run contrary to the results of previous studies, perhaps because electronic documentation systems are a core component of the healthcare process now, and they are very complex diverse systems. One nurse responded that “If a student cannot effectively document or find information on their patients in the EMR, they may jeopardize the care of their patients”. Therefore, the usefulness of the system is that it is critical in the care of patients.

## 6. Limitations

Our work is subject to several limitations. One limitation of our work is that we do not assess the specific system(s) used by participants, and the design and ergonomics of these systems could affect the behavioral factors examined in this work. Design considerations could consider taxonomies used for clinical narratives, disease classifications, and more. Ergonomics could affect nurses’ physical comfort using the system and effectiveness at completing work quickly. In future work, we are interested in examining this consideration in more detail to see the magnitude of its effect on nurses’ behaviors. Another important limitation is that our models did not consider the age of participants. Since participants only provided their age range rather than a specific value, the granularity of the data did not support a more sophisticated analysis. Relatedly, although the ages of the participants spanned a wide range, it is also important to note that some of the older participants may not have received training on computer skills in their formal nursing education, as the technology was not as widespread at the time. However, assessing the effects of age is an interesting potential area for future work. In addition, another interesting factor to consider in future work is nurses’ degrees and clinical experience and how this affects their behaviors. While we did not capture these factors in our paper, they could be of interest to future research.

## 7. Conclusions

In order to explore nurses’ perceived ease of using electronic documentation systems, exploring the nurse’s self-efficacy in using the system is necessary. Nurses’ self-efficacy in using the electronic documentation system indirectly affects the perceived usefulness of the system through a mediator (i.e., the system’s perceived ease of use). In nursing care, patients’ outcomes are the most important concern. Nurses using electronic documentation systems are more likely to develop perceived ease of use by continued daily use of the system on the job and through the assistance of mentors. Training facilities need to be excellent, and nurses also should feel confident in their computer skills to optimize patient care. This is because all electronic documentation systems are different and can be customized for each organization [39], making formal training crucial during the use of the system in practice.

Our research suggests that key aspects of nursing education, namely training, general computer skills, and self-efficacy, affect nurses’ propensities to find electronic documentation systems both easy to use and useful in their daily work. As the use of these systems contributes positively to the completeness and legibility of records [6] as well as a reduction in errors [5], our study suggests that these trainings may serve as one antecedent for improving the quality of nursing care. Of course, computer skills represent just one aspect of the nursing repertoire, and they represent one important part of well-rounded nursing education. An interesting avenue for future work to explore is the quality of these trainings and the extent to which different types of trainings may affect perceived ease of use and perceived usefulness.

A possible implication of our study concerns nursing education and internships, which play a role in building the fundamental computer skills that later affect perceived ease of use of electronic documentation systems. Building nursing students’ self-efficacy in their computer skills is vital, as this has an enormous impact on their acceptance of electronic documentation systems. Future work may explore the role that this education plays in the acceptance of electronic documentation systems.

## Figures and Tables

**Table 1:** Constructs of proposed technology acceptance model (TAM), definitions, and research hypotheses.

Constructs	Definitions
Self-efficacy [21,24,25]	A nurse’s judgment of his or her own capability to use an electronic documentation system (e.g., EHRs, EMRs, etc.).
Training [17,21]	The extent to which educators train nursing students on practical skills and deliver knowledge related to developing a person’s specific competence. (Based on classes in nursing training, the depth, quantity, and quality of the training, and the attitude of educators towards EHR software.)
General computer skills [26,27]	The extent to which nurses feel comfortable interacting with basic computer software, such as word processing, spreadsheets, etc.
Perceived usefulness [11,25]	“The degree to which a person believes that using a particular system would enhance his or her job performance” [11,25]. (Based on his or her perceptions of future use on the job.)
Perceived ease of use [11,25]	“The degree to which a person believes that using a system would be free of effort” [11,25]. (Based on his or her perception of electronic documentation system’s ease of use.)
Behavioral intention to use [28]	A nurse’s personal assessment of their likelihood of using the system.

**Table 2:** Sample descriptive statistics.

Demographic Category	Characteristic	Total	Required to Use EHRs	
			Yes	No
Age range	18–25	12	12	0
	26–33	38	38	0
	34–41	32	31	1
	42–49	47	46	1
	50–57	67	66	1
	58–64	44	44	0
	Over 64	7	7	0
Gender identity	Male	16	16	0
	Female	231	228	3

**Table 3:** Descriptive statistics on survey responses.

*N*	Measure	Number of Items	Mean	Standard Deviation	Min–Max	Skew	Kurtosis	α
248	GCS	7	4.51	0.98	1.00–6.00	−0.84	0.39	0.88
248	SE	6	5.21	0.73	1.60–6.00	−1.26	2.36	0.80
248	T	4	4.63	1.17	1.00–6.00	−0.76	−0.11	0.89
248	PEOU	8	4.42	0.90	2.00–6.00	−0.48	−0.21	0.75
248	PU	10	4.58	1.08	1.00–6.00	−0.58	−0.17	0.93
248	ITU	3	5.79	0.53	1.00–6.00	−4.68	31.17	0.78

**Table 4:** Goodness of fit statistics.

Measure	Chi-Squared	GFI	RMSEA
General computer skills (GCS)	31.28	0.97	0.07
Self-efficacy (SE)	18.01	0.98	0.06
Training (T)	3.24	0.99	0.05
Perceived ease of use (PEOU)	53.39	0.95	0.08
Perceived usefulness (PU)	41.24	0.96	0.07
Behavioral intention (ITU) (just-identified)	-	-	-

**Table 5:** Properties of items included under each construct.

Construct	Mean	SD
**General Computer Skills (GCS)**		
GCS2: I feel comfortable using word processing software (such as Word)	5.27	1.15
GCS3: I feel comfortable using spreadsheet software (such as Excel)	4.05	1.51
GCS4: I feel comfortable using database software (such as Access)	3.35	1.57
GCS5: I feel comfortable using presentation software (such as PowerPoint)	4.49	1.57
GCS6: I feel comfortable conducting Internet searches to find the information I need	5.52	0.90
GCS10: I feel intimidated if a conversation turns to computers	4.35	1.31
GCS11: I generally feel okay when trying something new on a computer	4.69	1.19
**Training (T)**		
T1: I received an appropriate amount of electronic documentation training in my nursing degree program to use an electronic documentation system effectively	3.06	1.67
T2: A specific person (or group) is available when needed for assistance with electronic documentation difficulties	4.69	1.28
T3: Specialized instruction and education concerning software about the electronic documentation system are available to me on the job	4.65	1.28
T4: Specialized programs or consultants about training on the electronic documentation system are available to me on the job	4.54	1.32
**Self-Efficacy (SE)**		
SE1: I currently have the necessary computer skills to use an electronic documentation system effectively	5.42	0.84
SE2: I feel confident finding patient information in the electronic documentation system	5.31	0.87
SE3: I expect to become more proficient in using electronic documentation system	5.35	0.96
SE5: I feel confident that I can use an electronic documentation system	5.49	0.77
SE6: I could complete my job using the electronic documentation system if there was no around to tell me what to do as I go	5.23	1.10
SE8: I could complete my job using the electronic documentation if I had used a similar system before this one to do the same job	4.53	1.37
**Perceived Ease of Use (PEOU)**		
PEOU2: The interface of an electronic documentation system is clear and easy to understand	4.25	1.41
PEOU3: It is easy for me to remember how to perform tasks using an electronic documentation	4.81	1.10
PEOU4: Interfacing with an electronic documentation system will require much mental effort	3.91	1.33
PEOU5: It is easy to get an electronic documentation system to do what I want it to	4.27	1.24
PEOU6: I find the electronic documentation system easy to use	4.61	1.21
PEOU7: I find it easy to get the electronic documentation system to do what I want it to do	4.36	1.26
PEOU9: My interaction with the electronic documentation system is clear and understandable	4.71	1.10
PEOU10: I think the electronic documentation system is simple to use in my consultation with patients	4.40	1.36
**Perceived Usefulness (PU)**		
PU1: Using an electronic documentation system improves my job performance	4.64	1.27
PU2: Using an electronic documentation system enhances my effectiveness on the job.	4.69	1.27
PU3: Using an electronic documentation system enables me to accomplish tasks more quickly	4.44	1.41
PU4: An electronic documentation system improves the quality of care that I could deliver	4.35	1.42
PU5: An electronic documentation system is useful in my job	5.06	0.99
PU7: Using the electronic documentation system allows me to have quick access to patient data	5.34	0.91
PU10: Using the electronic documentation system improved the quality of care	4.55	1.32
PU11: Using the electronic documentation system reduces the risk of error	4.79	1.18
PU12: An electronic documentation system improves my care of patients	4.41	1.33
PU13: An electronic documentation system makes it easier to care for patients	4.36	1.34
PU14: Using an electronic documentation system can effectively increase hospital credibility and image	4.65	1.38
PU15: Using an electronic documentation system reduces the amount of time in paperwork	4.48	1.47

**Table 6:** Correlation coefficients between measured variables.

	GCS	T	SE	PEOU
GCS				
T	0.35 **			
SE	0.29 **	0.27 **		
PEOU	0.45 **	0.53 **	0.48 **	
PU	0.34 **	0.42 **	0.32 **	0.66 **

Note: ** reflects statistical significance at the 0.05 level.

## Data Availability

The data presented in this study are available in the supplementary material.

## Supplementary Materials

The following is available online at https://www.mdpi.com/2039-4403/11/1/2/s1, Survey Data.

## Appendix A

**Table A1:** Excluded items.

Construct
**General Computer Skills (GCS)**
GCS1: I feel comfortable using email.
GCS7: I consider myself to be an experienced computer user.
GCS8: I feel fairly confident when working with computers.
GCS9: I am often unsure of what to do when using a computer.
GCS12: Learning about computers is essential for nurses working in today’s health services.
GCS13: I expect to use computers in many ways in nursing practice.
GCS14: All nurses should learn something about computing as part of their coursework.
**Training (T)**
T5: I have had enough training to use an electronic documentation system effectively
T6: I learned most of what I know about the electronic documentation system from colleagues on the job.
T7: I learned most of what I know about the electronic documentation system from using it on the job.
T8: I received an appropriate amount of electronic documentation training in my job to use an electronic documentation system effectively.
T9: Learning how to use the necessary electronic documentation features takes too much time.
T10: Learning how to use the electronic documentation system was easy for me since I had electronic documentation system training.
**Self-Efficacy (SE)**
SE4: I need to become more proficient in using an electronic documentation system.
SE7: I could complete my job using electronic documentation if I had never used a system like it before
**Perceived Ease of Use (PEOU)**
PEOU1: The electronic documentation system is easy to operate and navigate.
PEOU8: I find that interacting with an electronic documentation system demands much care or attention.
PEOU 11: I have to find ways to work around the electronic documentation system when it will not do what is needed.
**Perceived Usefulness (PU)**
PU6: Using an electronic documentation system is helpful in assisting the collection and analysis of patient data.
PU8: Using the electronic documentation system facilitates communication of information between various care providers.
PU9: Using the documentation system prevents duplication of examinations.
